# Three-Phase Equilibrium Calculations of Water/Hydrocarbon/Nonhydrocarbon Systems Based on the Equation of State (EOS) in Thermal Processes

**DOI:** 10.1021/acsomega.1c04522

**Published:** 2021-12-08

**Authors:** Xuesong Ma, Shuhong Wu, Gang Huang, Tianyi Fan

**Affiliations:** † Research Institute of Petroleum Exploration and Development, CNPC, 20 Xueyuan Road, Beijing 100083, China; ‡ China University of Petroleum (Beijing), 18 Fuxue Road, Beijing 102249, China

## Abstract

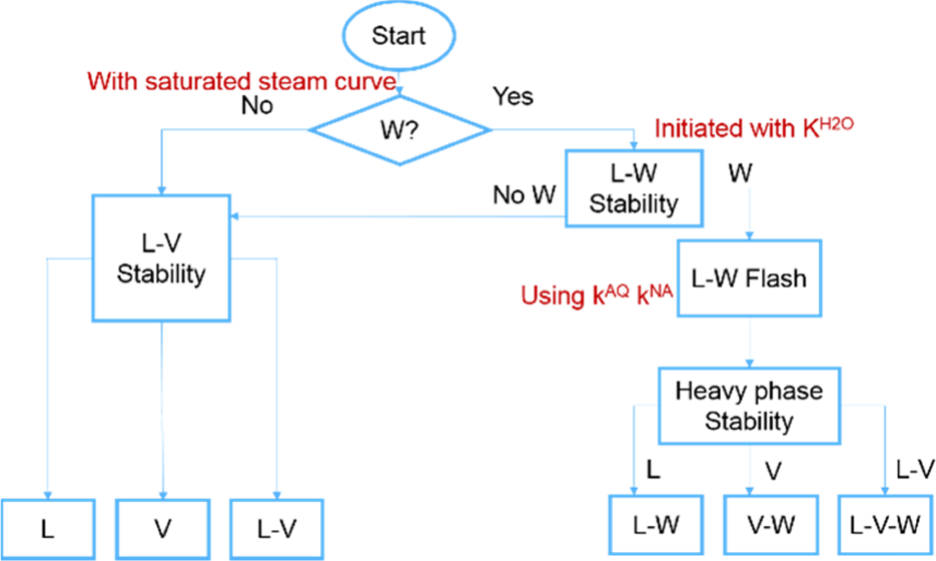

A simple and novel approach is proposed to represent the mutual solubility of water and hydrocarbon components based on equations of state at high temperatures in thermal recovery processes. Sϕreide and Whitson modifications are applied to the Peng–Robinson (PR) equation of state (EOS) so that all components, including the water component, can exist in all phases, reasonably representing gas solubility in water and water solubility in hydrocarbon phases. We propose an algorithm to assign binary interaction parameters (BIPs) for aqueous and nonaqueous phases. The water vapor pressure helps select initial *K*-values for stability analysis so that the aqueous phase can be split out first if present. The algorithm is tested by a wide range of variations in pressure, temperature, and composition. The results show the robustness of the algorithm and the effects of temperature and overall water mole fraction on phase behaviors in steam flooding processes.

## Introduction

Water is present either as initial water or as injection water and hence in contact with hydrocarbon phases in equilibrium in reservoirs. During the production of heavy oils often involved with three phases including oil, gas, and water, the *K*-value method is adopted in commercial simulators (Eclipse Technical Description 2013; STARS 2015; Intersect 2018) to predict phase behaviors in thermal processes (steam flooding). However, this method is not thermodynamically consistent because *K*-values are not of thermodynamic characteristics.^1^ Due to this drawback, the *K*-value method is competent for the performance of production where the *K*-values are weak functions of composition.^2,3^ However, especially for more complicated EOR methods such as Expanding Solvent Steam-Assisted Gravity Drainage (ES–SAGD), the *K*-value method is not accurate enough to represent the thermodynamic properties of three-phase fluid mixtures since it is only a function of temperature and pressure. In addition, the *K*-value method does not consider the mutual solubility of water and hydrocarbon, which can affect calculations of viscosity, density, and other thermodynamic properties. However, as the temperature increases, the solubility of water in hydrocarbon phases and the solubility of hydrocarbon in the aqueous phase are significantly increased.^4,5^ Several authors have investigated water solubility in hydrocarbon phases at high temperatures. Griswold and Kasch^6^ presented the concept of water/oil solubility at high temperatures. They indicated that water solubility in 54.3°API naphtha is 16.18 mol % at 431.67°F, water solubility in 42°API kerosene is 34.97 mol % at 507.2°F, and water solubility in 29.3°API oil is 43.44 mol % at 537.8°F. Glandt and Chapman^7^ showed that water solubility in oil is up to 33.3 wt %.^8−18^

In commercial simulators, Henry’s law is used to calculate the solubility in the aqueous phase.^19−21^ The cubic equation of state (EOS), meanwhile, is used to represent the fugacity coefficients in the nonaqueous phases. Two different formulations constitute an inconsistent algorithm for phase equilibrium calculations. Also, statistical associating fluid theory (SAFT) has also been proposed to represent the phase behavior of mixtures containing the aqueous phase. But, it requires knowledge of corresponding molecular structures. Thus, it is not suitable for industrial applications. Huron and Vidal^22^ presented a nonsymmetrical model on the basis of the SRK equation, but the accuracy of the model is not high enough.^23^ Chapman^24^ developed a sum of three Helmholtz energy terms based on the EOS called SAFT (statistic associating fluid theory). Kontogeorgis^25,26^ proposed the association of compressibility factor based on cubic models called cubic plus association (CPA). Li and Firoozabadi^27^ described the cross association between non-water components and water in the framework of perturbation theory. Li^28^ noted that SAFT and CPA models usually exhibit good performance for standalone calculations, but they are not time-efficient when used for numerical simulations.

Another approach for equilibrium calculations for thermal compositional simulation is based on equations of state. Phase behavior representation of water-containing mixtures using the equation of state remains a challenge due to the nonideality brought by strong hydrogen bonds among water molecules. A good reproductivity of compositions in nonaqueous phases can be achieved, but the calculated composition for the aqueous phase is inconsistent with the experimental data by several orders of magnitude since slight amounts of multiple components usually exist in the water phase.

An accurate prediction of the phase behavior of mixtures using the equation of state depends on appropriately chosen binary interaction parameters, mixing rules, and the α function for pure substances. The most important modification of the original EOS for water/hydrocarbon systems is the introduction of two sets of binary interaction parameters for constant *a*. Whitson and Brule^29^ verify the accuracy of simultaneous application of aqueous- and nonaqueous-phase interaction coefficients for mutual-solubility predictions of binaries and natural gas/water/brine mixtures, suggesting that the modification is warranted. Li and Yang^30^ developed a new binary interaction parameter correlation to determine mutual solubility between CO_2_ and water. The polynomial temperature-dependent BIP correlation in the aqueous phase predicts more accurately than the exponential BIP correlation. Composition- and density-dependent mixing rules have also been proposed for modifying cubic EOSs for water/hydrocarbon systems. Panagiotopoulos and Reid’s^31^ linear composition-dependent mixing rule has received considerable interest. Unfortunately, it violates several fundamental thermodynamic conditions. To improve the predictions of water vapor pressure and water solubility in the nonaqueous phases, Peng and Robinson proposed a modified α function for water, which can be used for 284.8 < *T* < 466.1 K. Sϕreide and Whitson^32^^32^ replaced the α function with a new correlation. It can predict water vapor pressures for 288.2 < *T* < 598.15 K. Li and Yang^30^ developed a modified α function that is able to more accurately reproduce the water vapor pressure in the full temperature range of 273.15–647.10 K.

Because of a more extensive temperature range, we adopt the α function from Li and Yang.^30^ Due to the simplicity and reasonable prediction of mutual solubility between light hydrocarbons and water, we adopt BIP correlations from the Sϕreide and Whitson^32^ model to match the mutual solubility of hydrocarbon and water. Mohebbinia et al.^33,34^ also adopted Sϕreide and Whitson modifications to represent gas and water mutual solubility. However, the method of phase identification has not been mentioned, which is required in advance to determine the different sets of BIPs between hydrocarbon components and water in the aqueous phase and nonaqueous phases. We predict whether an aqueous phase is present by virtue of the water vapor pressure curve. If an aqueous phase may be present, the equilibrium calculation is performed first with a nearly pure water phase as a trial phase for the first stability analysis to obtain phase fractions and compositions of the hydrocarbon-rich phase and the aqueous phase.

The sequential algorithm combining phase stability analysis and phase splitting is used.^35−38^ First, according to the overall composition, the stationary point method is used at the present temperature and pressure to determine how many phases exist. If the mixture is unstable, another phase is added and an ensuing flash calculation provides phase fractions and phase compositions. The heavier phase with a heavier molecular weight is applied to the second stability analysis.^39^ If the heavier phase is stable, then two phases are in equilibrium; if it is not stable, another phase is added and a three-phase flash calculation is required.^40^

The paper is structured along the following lines. First, we present the binary interaction parameter correlations from Sϕreide and Whitson^32^ and the α function for water from Li and Yang,^30^ followed by the characteristic of the water vapor pressure curve. Then, the selection of initial guesses for stability analysis is presented, followed by the gas–oil–water three-phase equilibrium algorithm. The results of our method compared with other models to represent water/hydrocarbon mutual solubility are presented. Several examples taken from the literature are tested, including the construction of phase envelopes in two- and three-phase regions. And this section also illustrates variations in phase distribution as an overall water mole fraction and temperature increase. Finally, the main results and conclusions are summarized.

### Modified PR EOS for the Aqueous Phase

Phase properties are calculated by PR EOS in our work, and all components, including water, can exist in all phases. Water is not a separate phase. Li and Yang^30^ developed a modified α-term to more accurately reproduce the water vapor pressure in the full temperature range of 273.15–647.10 K.
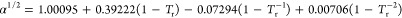
1where *T*_r_ is the reduced temperature for water.

According to Sϕreide and Whitson,^32^ two sets of binary interaction parameters (BIPs) between hydrocarbon components and water are proposed for nonaqueous phases and the aqueous phase. As a result, two different attraction terms in the EOS are calculated as a function of their respective BIPs.
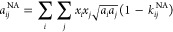
2
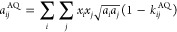
3Sϕreide and Whitson^32^ suggested that a constant BIP for the nonaqueous phase and a temperature-dependent BIP for the aqueous phase were found adequate to match experimental mutual solubility data with reasonable accuracy. BIP constants for the nonaqueous phase are listed in Table 1, and a constant equal to 0.5 is applied for hydrocarbon/water binaries not listed in Table 1.

**Table 1:** Nonaqueous Phase Binary Interaction Parameters between Water and Various Components

component	*K*_iw_^NA^
C_1_	0.4855
C_2_	0.4920
C_3_	0.5525
*n*-C_4_	0.5091
N_2_	0.4778
CO_2_	0.1896

Sϕreide and Whitson^32^ proposed four correlations to calculate BIPs in the aqueous phase between hydrocarbon/brine, CO_2_/brine, N_2_/brine, and H_2_S/brine. Recently, Li and Yang^30^ proposed the BIP correlation for CO_2_/brine. This work adopts the BIP correlation for CO_2_/brine from Li and Yang,^30^ while BIP correlations for hydrocarbon/brine, N_2_/brine, and H_2_S/brine are taken from Sϕreide and Whitson.^32^
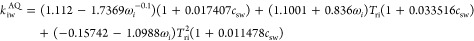
4
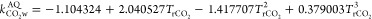
5
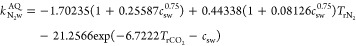
6
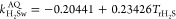
7where *T*_ri_ is the reduced temperature, *c*_sw_ is the salinity of brine, and ω_*i*_ is the acentric factor for each component.

Compared with the original PR EOS formulation, BIP and α function modifications to PR EOS provide a more accurate representation of the true multiphase equilibrium than the original PR EOS approach. This method entails the identification of phase types upfront, so that different sets of BIPs can be applied to nonaqueous and aqueous phases.

In steam flooding processes, overall mixture compositions in reservoirs are gradually dominated by the injection steam, i.e., the water component. For stability analysis and phase-splitting calculations involving the aqueous phase, if the water component accounts for no less than 50%, the mixture is taken as waterlike, and binary interaction parameters (BIPs) for the aqueous phase are used for the evaluation of component fugacity coefficients. If the mole fraction of the water component is less than 50%, binary interaction parameters (BIPs) for the nonaqueous phase are chosen for the phase.

### Vapor Pressure

In thermal settings, a key point is that the saturated steam curve can be used to guide the selection of the trial phase for stability analysis. This distinctive characteristic for hydrocarbon–water systems is sensitive to different phase states of the systems. If the reservoir pressure is lower than the water vapor pressure at its current temperature, the aqueous phase is unlikely to exist. Then, stability analysis is started with Wilson’s correlation and its inverse as initial guesses. If the reservoir pressure is higher than the water vapor pressure, the aqueous phase can be identified by K_**2**_^HO^ as an initial guess for stability analysis. The rationale behind the method is that the aqueous phase may only exist at pressures higher than water vapor pressure. If the first stability analysis result shows that the aqueous phase is not present, Wilson’s correlation and its inverse are used as initial guesses again for the stability analysis.

We use eq 8 to calculate water vapor pressure^41^


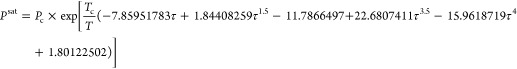
8

where , *T*_c_ is the critical temperature, and *P*_c_ is the critical pressure.

Note that the validation of the above correlation corresponds to the temperature range between 273.16 and 647.14 K. As Whitson and Brulé^29^ point out, the critical temperature defines the temperature above which any gas/liquid mixture cannot coexist, regardless of pressure. Similarly, the critical pressure defines the pressure above which liquid and vapor cannot coexist, regardless of the temperature. At the critical point, the vapor and liquid phases can no longer be distinguished, and their intensive properties are identical. So, the overall mixture contains no aqueous phase when the temperature is higher than 647.14 K.^42−45^

## Initial Guess for Stability Analysis

In the stability analysis, the local minimum of the tangent plane distance function (TPD) is strongly dependent on the initial guess of the trial phase composition, which can directly affect equilibrium ratios *K* converged at the end of stability analysis. Improper initial guesses may miss some stationary points and fail to detect phase instability. Furthermore, stability analysis provides initial *K*-values for phase splitting calculations. Inappropriate initial *K*-values may result in the failure of flash calculations. For vapor–liquid equilibrium systems, {*K*_Wilson_} can provide good initial guesses for stability analysis.^46^ However, for liquid–liquid equilibrium systems, {*K*_Wilson_} becomes unreliable and may lead to failures in detecting instability. Our work adopts the stationary point location method from Michelsen^35^ with an initial guess suggested by Li and Firoozabadi^47^ for the second stability analysis to overcome the setback in equilibrium calculations of more than one liquid phase.^48−51^

We use two types of multiple initial *K*-value estimates for the first stability analysis. If the aqueous phase is not present, the vapor–liquid mixtures comprised of hydrocarbon components are tested by Wilson’s correlation eq 9 and its inverse as initial guesses. Otherwise, stability analysis is initiated by a nearly pure water phase as the trial phase when the aqueous phase exists in equilibrium.
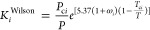
9where *T*_c*i*_, *p*_c*i*_, and ω_*i*_ are the critical temperature, critical pressure, and acentric factor of component *i*.

In a nearly pure aqueous phase, the fraction of water component is 99 mol %.
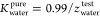
10And the other (*N*_c_ – 1) components equally share the remaining 1 mol % in the trial phase.
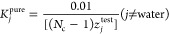
11When the aqueous phase is present in the feed composition, the first stability analysis is started with *K*^pure^ (water accounts for 99% in the trial phase). If the mixture is unstable, the converged nontrivial *K*-values from the first stability analysis are used as the initial *K*-values for the two-phase splitting calculation, so that the aqueous phase can be split out of the mixture first. Also, only after the aqueous phase is distinguished from nonaqueous phases can we distribute two types of BIPs for the aqueous phase and nonaqueous phases.

In the three-phase flash calculations, the second stability analysis is performed to check whether the physical solution of the first flash is stable in the two-phase state. According to Li and Firoozabadi,^47^ the heavier phase (with higher molecular weight) is selected as the test phase because the aqueous phase is mainly composed of water, and accordingly, the aqueous phase is not the heavier phase. The hydrocarbon phase is naturally chosen to be tested as the test phase. To overcome the intrinsic setback of the stationary point method, we use multiple initial estimates in the second stability analysis. Li and Firoozabadi^47^ suggested that the initial composition of one component is 90% and that the other components equally share the remaining 10 mol % of the trial phase.
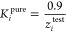
12
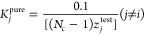
13

There are *N*_c_ sets of initial guesses of {*K*_*j*_^pure^} because every component has the opportunity to be assigned 90 mol %.

For the second stability analysis, the initial guesses in our work are
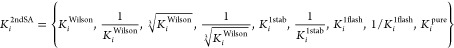
14*K*_*i*_^1stab^ represents the converged nontrivial solution of the first stability analysis corresponding to the lowest TPD and *K*_*i*_^1flash^ is the *K*-values from the two-phase splitting calculation. The number of initial guesses in the second stability analysis is *N*_c_ + 8. All of these estimates may detect phase instability.

## Algorithm for Gas–Oil–Water Phase Equilibrium Calculation

Stability analysis turns out to be a heavy burden in the whole process of multiphase equilibrium calculations because of the existence of multiple initial estimates for trial phases. We propose an efficient and reliable scheme for phase behavior calculations, taking advantage of characteristic behaviors of water/hydrocarbon mixtures.

If the current pressure is lower than the water vapor pressure, water is not present. The stability analysis is initiated with Wilson’s correlation and its inverse as trial phases. The result may be single-phase, either as a liquid or as vapor, or a two-phase mixture that requires a subsequent two-phase splitting calculation. If the reservoir pressure is higher than the water vapor pressure, the aqueous phase may exist in equilibrium. Then, the stability analysis is started with a nearly pure water phase as the initial estimate. If TPD > 0, the aqueous phase does not exist. Then, the same strategy is adopted as the previous case when pressure is below the steam pressure. If the stability test result shows that the mixture is not stable, the converged nontrivial *K*-values from the nearly pure water trial phase are used to initiate the two-phase splitting calculation so that the aqueous phase is split out as the second phase. Thus, the identity of each phase in the splitting process is specified and different BIPs for the aqueous phase and the hydrocarbon phase are assigned for each phase. The second stability analysis is applied to the heavier phase, i.e., the hydrocarbon phase. The trial phases are related to Wilson’s correlation, its inverse, its cubic, the inverse of cubic, and nearly pure phases consisting of certain components except for water. If the second stability analysis shows that the heavier phase is unstable, then the *K*-values corresponding to the lowest TPD in the second stability analysis are selected to initiate the three-phase splitting calculation. BIPs for the aqueous phase are assigned for the third phase, and BIPs for nonaqueous phases are assigned for the first two phases. If the result of the second stability analysis is stable, the system is a two-phase mixture. The main steps involved in the gas/oil/water three-phase equilibrium calculations are as follows:

- Step 1: Evaluate the water vapor pressure at the current temperature by eq 8 to determine whether an aqueous phase is present. If an aqueous phase is not present, go to step 3; if an aqueous phase may be present, go to step 2.
- Step 2: Initiate the first stability analysis with a nearly pure water phase as the trial phase. If the result shows that the mixture is stable, then the mixture contains no aqueous phase, and so go to step 3. If the result shows that the mixture is unstable, go to step 4.
- Step 3: Initiate the stability analysis with Wilson’s correlation and its inverse. If two TPDs corresponding to nontrivial solutions are both positive, the mixture is single-phase; if no less than one initiate estimate leads to a negative TPD, then the converged *K*-value corresponding to the lowest TPD is selected to initiate a two-phase splitting calculation and obtain a liquid–vapor result.
- Step 4: Perform the two-phase splitting calculation with the converged nontrivial *K*-values from the first stability analysis initiated with a nearly pure water phase. BIPs for the aqueous phase are assigned for the second phase (aqueous phase), and the nonaqueous phase BIPs are assigned for the first phase (hydrocarbon phase) when computing component fugacities in each phase.
- Step 5: The second stability analysis is applied to the hydrocarbon phase. The initial guesses are as in eq 14. If the hydrocarbon phase is stable, the mixture is two-phase. Otherwise, go to the next step.
- Step 6: Perform a three-phase splitting calculation initiated with the converged nontrivial *K*-values corresponding to the lowest TPD from the second stability. Aqueous phase BIPs are assigned for the third phase (aqueous phase), and nonaqueous phase BIPs are assigned for the first two phases (hydrocarbon phases).

One advantage of our work is that no matter whether vapor–liquid phases or liquid–liquid phases are identified by the second stability analysis, it is impossible to result in the existence of two vapor phases in equilibrium after the three-phase splitting calculation, which ensures the reasonable results. If the result of the first stability analysis initiated with a nearly pure water phase as the trial phase shows that the mixture is stable, then the aqueous phase is not present. The system is only comprised of hydrocarbon phases. Wilson’s correlation and its inverse are sufficient to identify whether the hydrocarbon mixture is two-phase or single-phase.

**Figure 1:**
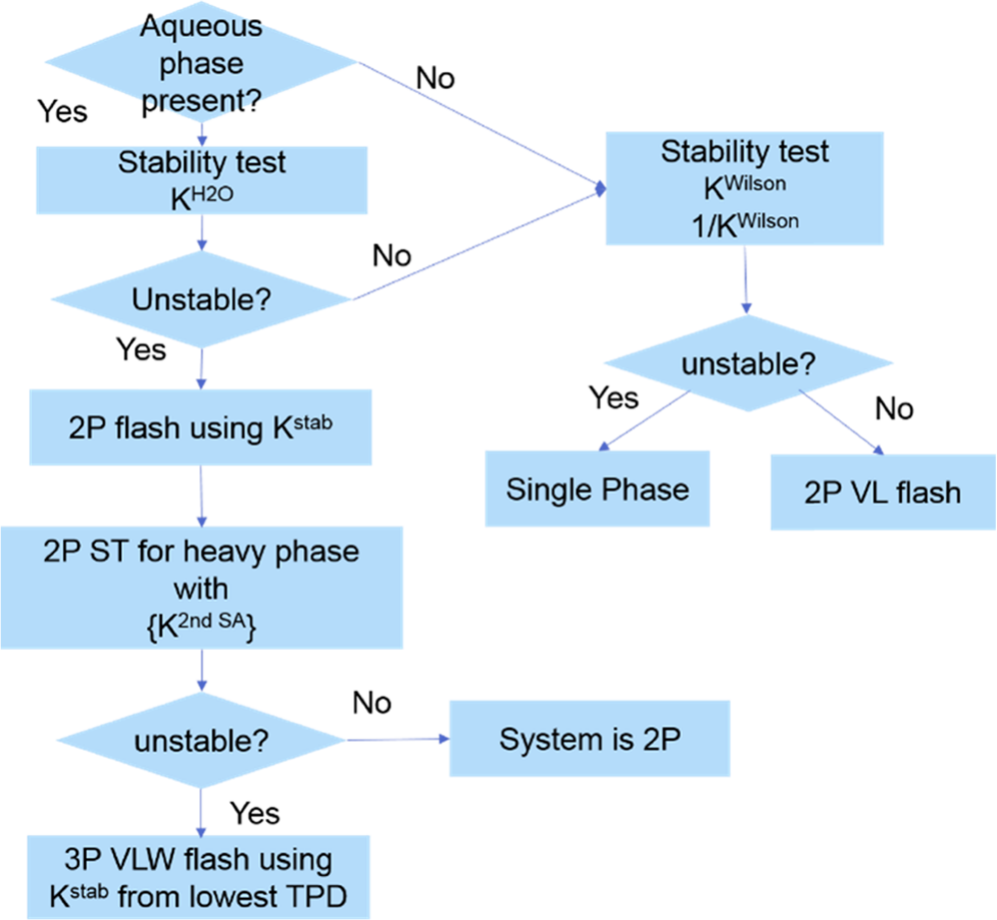
Algorithm for gas/oil/water three-phase equilibrium calculations.

Because equal fugacity is a necessary but not sufficient condition for phase equilibrium, a poor initial guess corresponding to the lowest Gibbs free energy may lead to an erroneous flash solution. In most cases, the best initial estimates for flash calculation are *K*-values corresponding to the lowest TPD in the stability analysis, but it is not the whole picture. If local minima and the global minimum have close Gibbs free energy, and more than one stationary point is located corresponding to negative TPDs, then the *K*-values corresponding to the lowest TPD may cause erroneous flash results. If this happens, the *K*-values corresponding to the next lowest TPD from stability analysis are tried to start the ensuing flash calculation (Figure 1).

## Results and Discussion

### Mutual Solubility

**Figure 2:**
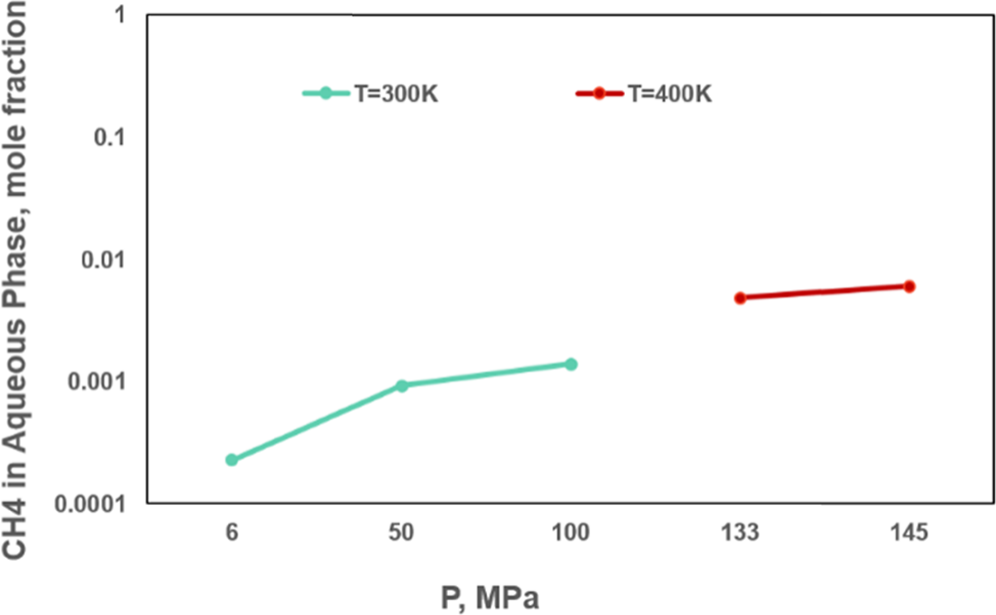
Hydrocarbon solubility in the aqueous phase calculated by our method in three-phase regions at different temperatures.

**Figure 3:**
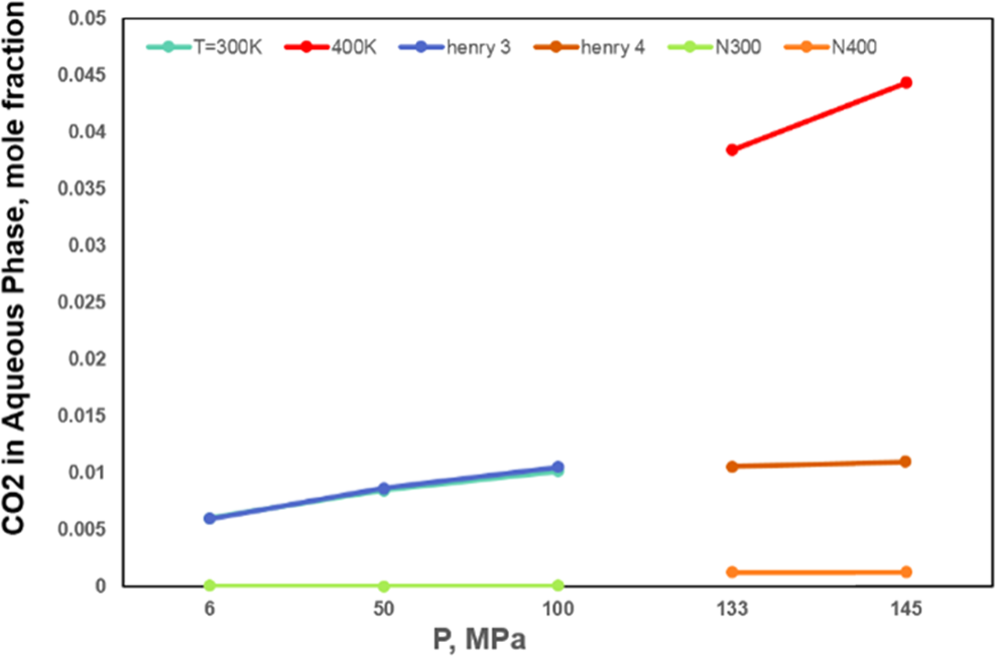
Compared with Henry’s law and original PR EOS formulation, CO_2_ solubility in the aqueous phase calculated through our work in three-phase regions at different temperatures.

**Figure 4:**
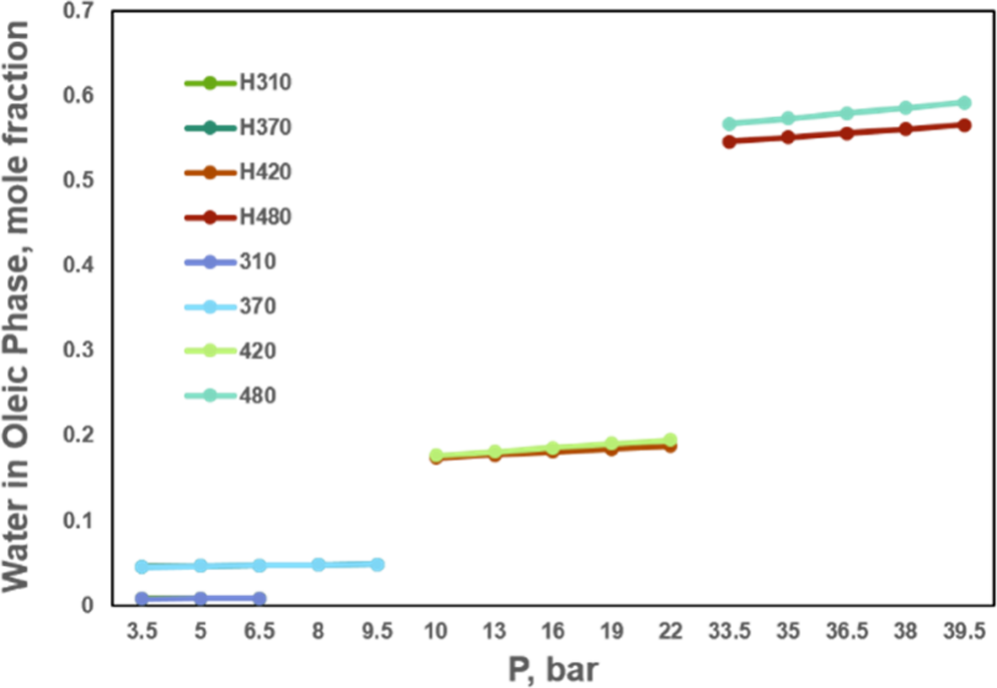
Changes in water solubility in the oleic phase with temperatures in three-phase regions calculated through both our method and Henry’s law.

To quantify the mutual solubility of water and hydrocarbons, we examine water solubility in the oleic phase and hydrocarbon solubility in the aqueous phase at different temperatures in the three-phase region. Compared with Henry’s law, hydrocarbon solubility in the aqueous phase is examined through a synthetic quaternary mixture taken from Mohebbinia et al.,^33^ which consists of CO_2_, methane, normal hexadecane, and water. The fluid properties are listed in Table 2, and BIPs for nonaqueous phases are provided in Table 3. BIPs for the aqueous phase are calculated according to eqs 4 and 5. Figure 2 shows that methane solubility in the aqueous phase increases to 1% with temperature on the order of 10^–2^. Under Henry’s law, there is no gas solubility in the aqueous phase. Figure 3 illustrates that the CO_2_ solubility in the aqueous phase calculated through our method is significantly higher, compared with Henry’s law and original PR EOS formulation. We evaluate water solubility in the oleic phase through our method in comparison with Henry’s law. This example uses a synthetic oil mixture taken from Luo and Barrufet,^52^ which consists of 0.25 water, 0.15 pseudocomponent 1 (PC1), 0.15 pseudocomponent 2 (PC2), 0.2 pseudocomponent 3 (PC3), and 0.25 pseudocomponent 4 (PC4).^53^ The component characteristics are described in Table 10. Since the BIPs for the nonaqueous phases used in Luo and Barrufet^52^ are not given, we use the BIPs between water and pseudocomponents for the nonaqueous phases from Zhu and Okuno,^54^ described in Table 11. The pressure ranges are selected where the gas/oil/water three phases exist at the given temperatures. Simulation results are shown in Figure 4. With temperature elevated, the solubility of water in the oleic phase increases up to 0.6 at 480K so that the solubility is non-negligible in a thermal simulation process. There is an excellent agreement of water solubility in the oleic phase between Henry’s law and our method at moderate temperatures and low pressures. However, it turns out that the gas/oil/water three-phase flash using Henry’s law leads to a lower water solubility at elevated temperatures than our work, and then it predicts a lower oleic production rate in a thermal simulator. So Henry’s law is suitable for low temperatures but not for high temperatures in thermal recovery processes.

**Table 2:** Component Properties for the Synthetic Quaternary Fluid

component	mol %	MW (g/mol)	*T*_c_ (K)	*P*_c_ (bar)	ω
CO_2_	75.0	44	304.2	73.8	0.225
CH_4_	2.5	16	190.6	46.0	0.008
*n*-C_16_	2.5	226	717.0	14.2	0.742
H_2_O	20.0	18	647.3	220.5	0.344

**Table 3:** Nonaqueous Phase Binary Interaction Parameters for the Synthetic Quaternary Fluid

BIP	CO_2_	CH_4_	*n*-C_16_	H_2_O
CO_2_	0			
CH_4_	0.1000	0		
*n*-C_16_	0.1250	0.0780	0	
H_2_O	0.1896	0.4850	0.5000	0

### Power of our Algorithm

Two examples are presented to validate two-phase splitting calculation and three-phase splitting calculation in comparison with results from the dissertation of Varavie^4^ and CMG-WinProp. The algorithm adopted in the dissertation of Varavie is a free-water three-phase flash, under the assumption that water is a separate phase. The routine in CMG-WinProp employs Henry’s law to represent the aqueous phase. The two-phase equilibrium calculation is carried out at a pressure of 34.5 bar and a temperature of 333.15 K. The input data are listed in Tables 4 and 5, and the results of the two-phase equilibrium are shown in Table 6. The agreement of two-phase equilibrium calculation among our works and other methods is excellent. There is no hydrocarbon solubility in the aqueous phase since the feed mixture contains no hydrocarbon gas at the test temperature. It is shown that water solubility calculated by our method is slightly higher than those from two other methods, which validates a more accurate representation of water solubility in hydrocarbon-rich phases provided by our approach. The pressure and the temperature of the three-phase splitting calculation are performed at 13.79 bar and 366.5 K. Tables 7, 8, and 9 present the input data and the three-phase splitting results. It can be observed that the concentration of the light hydrocarbon component in the aqueous phase calculated by our method is higher than that by the two other approaches. This is further validation of involving gas solubility in the aqueous phase in our work. Furthermore, the concentration of the water component in the oleic phase calculated by our work is much higher than those obtained with the other three-phase flash methods; a similar conclusion can be drawn for the vapor phase.

**Table 4:** Component Properties for Two-Phase Equilibrium Calculation

component	MW (g/mol)	*T*_c_ (K)	*P*_c_ (bar)	ω
H_2_O	18	647.3	220.47	0.344
C_6_	86	507.5	32.89	0.275
C_10_	134	622.1	25.34	0.444
C_15_	206	718.6	18.49	0.651

**Table 5:** Nonaqueous BIPs for Two-Phase Equilibrium Calculation

BIP	H_2_O	C_6_	C_10_	C_15_
H_2_O	0			
C_6_	0.48	0		
C_10_	0.48	0.002866	0	
C_15_	0.48	0.010970	0.002657	0

**Table 6:** Results of the Two-Phase Equilibrium Calculation, Compared with Those of Varavei and WinProp

		phase 1, mol %			phase 2, mol %		
component name	overall mol %	this work	Varavei	WinProp	this work	Varavei	WinProp
C_6_	10.0	16.634	16.636	16.636	0	0	0
C_10_	20.0	33.269	33.272	33.273	0	0	0
C_15_	30.0	49.903	49.908	49.909	0	0	0
H_2_O	40.0	0.194	0.184	0.1809	100	100	100
phase fraction, %	60.116	60.111	60.109	39.884	39.889	39.891	

**Table 7:** Component Properties for Three-Phase Equilibrium Calculation

component	MW (g/mol)	*T*_c_ (K)	*P*_c_ (bar)	ω	overall mol %
H_2_O	18	647.3	220.47	0.344	10.0
C_1_	16	190.6	46.00	0.008	10.0
C_6_	86	507.5	32.89	0.275	20.0
C_10_	134	622.1	25.34	0.444	40.0
C_15_	206	718.6	08.49	0.651	20.0

**Table 8:** Nonaqueous BIPs for Three-Phase Equilibrium Calculation

BIP	H_2_O	C_1_	C_6_	C_10_	C_15_
H_2_O	0				
C_1_	0.485	0			
C_6_	0.48	0	0		
C_10_	0.48	0	0.002866	0	
C_15_	0.48	0	0.010970	0.002657	0

**Table 9:** Results of Three-Phase Equilibrium Calculation, Compared with Those of Varavei and WinProp

	phase 1, mol %			phase 2, mol %			phase 3, mol %		
component name	this work	Varavei	WinProp	this work	Varavei	WinProp	this work	Varavei	WinProp
**C**_**1**_	3.961	3.984	3.976	90.8347	91.1740	91.1904	0.0004	0.0002	0.0002
**C**_**6**_	13.372	13.374	13.375	2.8802	2.8763	2.8793	0	0	0
**C**_**10**_	27.329	27.331	27.333	0.2770	0.2779	0.2770	0	0	0
**C**_**15**_	54.717	54.718	54.724	0.0119	0.0119	0.0119	0	0	0
**H**_**2**_**O**	0.622	0.593	0.592	5.9951	5.6599	5.6414	99.9587	99.99	99.99
**phase fraction, %**	73.102	73.0996	73.0915	7.813	7.7738	7.7796	19.085	19.1266	19.1289

### Effects of Temperature and Overall Water Mole Fraction

**Figure 5:**
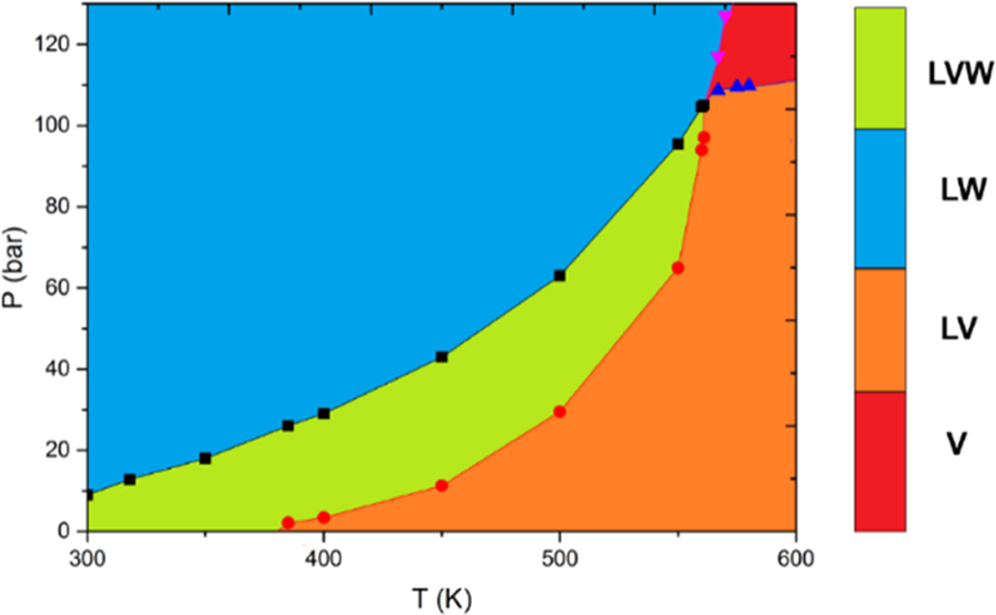
PT diagram for the water/heavy crude oil mixture. V is the vapor phase, L is the oleic phase, and W is the aqueous phase.

We test our algorithm across a large parameter space to validate the robust calculations of phase compositions in difficult cases. This case uses the mixture taken from Zhu and Okuno.^54,55^ The mixture consists of 50% pseudocomponent heavy crude oil and 50% water. The pure-component properties are listed in Table 10. The binary interaction parameters (BIPs) for nonaqueous phases are shown in Table 11, and the binary interaction parameters (BIPs) for aqueous phases are assigned through eq 4. Figure 5 shows the phase diagram of the water/heavy oil mixture. There are no convergence problems even in the vicinity of phase boundaries.

**Table 10:** Component Properties for Water/Heavy Oil Mixture

component	feed	MW (g/mol)	*T*_c_ (K)	*P*_c_ (bar)	ω
H_2_O	0.5	18.015	647.3	220.89	0.344
PC1	0.15	30.00	305.556	48.82	0.098
PC2	0.1	156.00	638.889	19.65	0.535
PC3	0.1	310.00	788.889	10.20	0.891
PC4	0.15	400.00	838.889	7.72	1.085

**Table 11:** Nonaqueous BIPs for Water/Heavy Oil Mixtures

BIP	H_2_O	PC1	PC2	PC3	PC4
H_2_O	0				
PC1	0.71918	0			
PC2	0.45996	0	0		
PC3	0.26773	0	0	0	
PC4	0.24166	0	0	0	0

**Figure 6:**
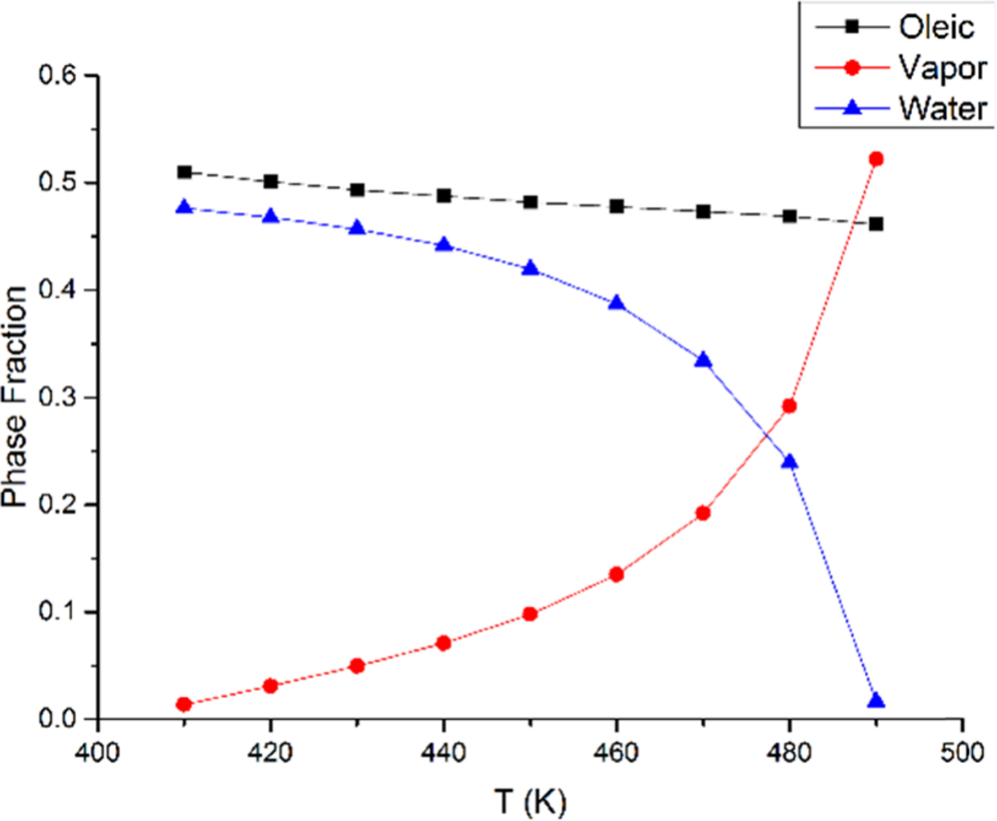
Phase distributions of oleic, vapor, and aqueous phases across the temperature range for water/heavy oil mixtures at 30 bar.

**Figure 7:**
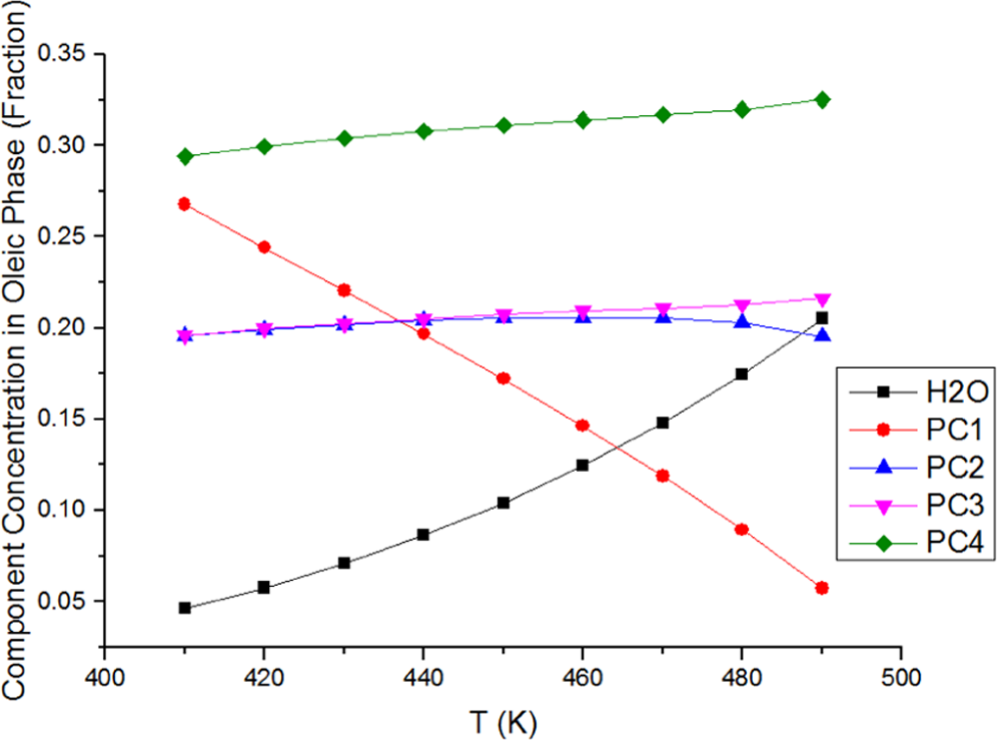
Changes in the oleic phase composition across the temperature range for water/heavy oil mixtures at 30 bar.

**Figure 8:**
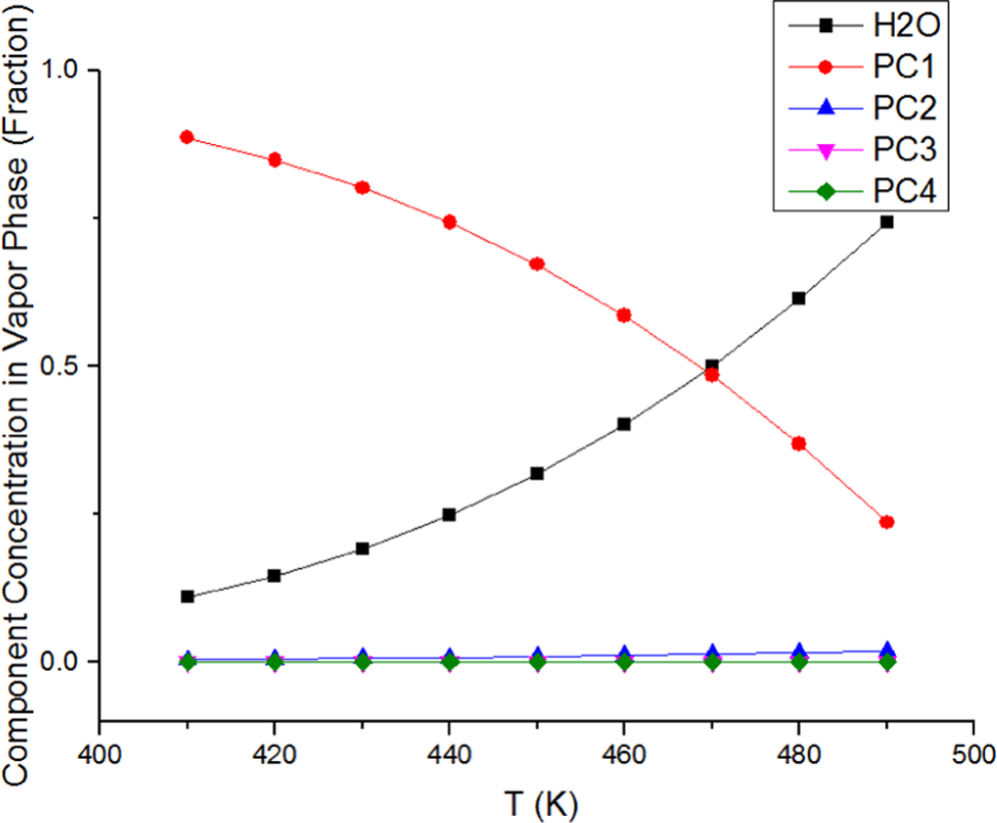
Changes in the vapor phase composition across temperature ranges for water/heavy oil mixtures at 30 bar.

Figure 6 indicates that phase fractions are sensitive to temperature. At 30 bar, the vapor phase fraction increases from 0 to 54%, and the aqueous phase decreases from 49% to 0 with the temperature elevated in the three-phase region. The reason behind this phenomenon is correlated with the variations in component distributions in each phase. As shown in Figures 7 and 8, the water concentration increases in both the oleic phase and the vapor phase as the temperature increases. Therefore, the aqueous phase fraction falls drastically. The aqueous phase is dominated by 99.9% water component, and so its composition is not shown here. The concentration of PC1 in the oleic phase and the vapor phase reduces significantly with the increasing temperature, but the other three components change slightly in both phases.

Another set of equilibrium calculations are performed on the water/heavy oil mixture to demonstrate the effect of overall water mole fraction in feed composition on phase distributions in the three-phase region. The flash calculations are performed at 15 bar, with variations in temperatures and overall water mole fractions. With increasing temperature, the oleic phase fraction decreases slightly; the vapor phase fraction increases, but the aqueous phase fraction drops. In the heating process, the water component in the aqueous phase and the light hydrocarbons in the oleic phase become volatile, and phase transitions occur with the elevated temperature. When the temperature is less than 390 K, the oleic phase transits into the vapor phase, and the aqueous phase seldom changes. The variations in both oleic and vapor phase fractions are less than 10% because the overall mole fraction of volatile hydrocarbon components in the feed mixture is small. However, if the temperature is over 390 K, the vapor phase fraction increases significantly mainly because of the noticeable decrease in the amount of the aqueous phase. The phase amounts change dramatically when the temperature is more than 390 K since the overall water mole fraction is larger than that of the light hydrocarbon component in the feed composition. As is well known, the boiling temperature of water is higher than that of the light hydrocarbon component, and that is why a significant decrease in the aqueous phase mole fraction occurs after the temperature reaches 390 K.

**Figure 9:**
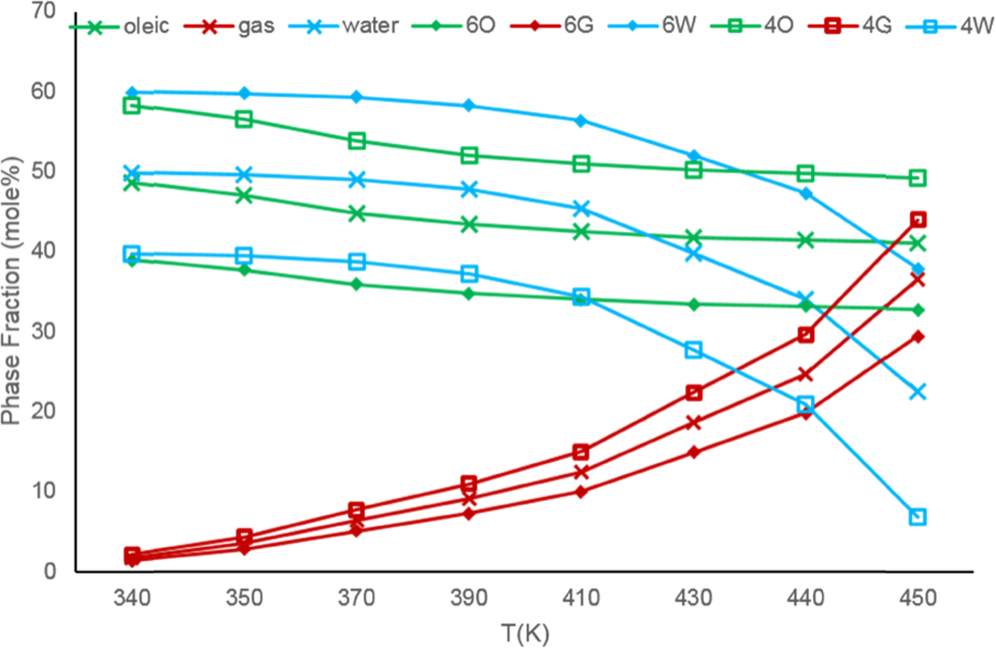
Phase distributions across variations in overall water fraction and temperature at 15 bar for water/heavy oil mixtures.

We test our method on the basis of a compositional range by combining different fractions of water with the above hydrocarbon mixture taken from Zhu and Okuno.^54,55^ The overall water mole fraction in the system is increased from 40 to 60%, to evaluate the effect of water mole fraction on phase distributions. The increase in overall water mole fraction, as is the case in steam flooding, shifts the vapor phase fraction curve toward higher temperatures. That is because water is introduced into the feed system in the form of vapor. With the addition of water, the oleic phase fraction drops, but the aqueous phase fraction mounts at a certain temperature (Figure 9).

## Conclusions

A simple but robust algorithm was developed based on the modified PR EOS to describe the gas/oil/water three-phase behaviors of water-containing systems. The water vapor curve is used to predict whether or not an aqueous phase may be present, and accordingly, one of the two sets of initial estimates is chosen to initiate the first stability analysis. We employ the initial estimates of the second stability analysis from Li and Firoozabadi.^47^ The developed algorithm is evaluated on a wide range of variations in temperature, pressure, and composition to validate the power of the algorithm.

A standard is stipulated that if the water component accounts for no less than 50%, the mixture is taken as waterlike and binary interaction parameters (BIPs) for the aqueous phase are used for evaluation of component fugacity coefficients; otherwise, BIPs for the nonaqueous phase are selected.

The mutual solubility of water and hydrocarbons caused by high temperatures is shown.

The robustness of the proposed algorithm is shown by several examples. Convergence is obtained without problems even close to the phase boundaries.

The effects of temperature and overall water mole fraction on phase distributions are displayed. As the temperature increases, the vapor phase fraction mounts; with the addition of water, the amount of oleic phase decreases. Specific variations are dependent on the feed composition and current conditions.

## Abbreviations

PR: Peng–Robinson
RK: Redlich–Kwong
EOS: equation of state
BIP: binary interaction parameters
EOR: enhanced oil recovery
SRK: Soave–Redlich–Kwong
CPA: cubic plus association
TPD: tangent plane distance function in stability analysis
SAFT: statistical associating fluid theory
ES–SAGD: expanding solvent steam-assisted gravity drainage

## Nomenclature

α: PR EOS attraction term
*T*_r_: reduced temperature
*c*_sw_: salinity of brine
*k*_*ij*_^NA^: BIP between components *i* and *j* in the nonaqueous phase
*k*_*ij*_^AQ^: BIP between components *i* and *j* in the aqueous phase
*a*_*ij*_^NA^: PR EOS energetic term for the nonaqueous phase
*a*_*ij*_^AQ^: PR EOS energetic term for the aqueous phase
K: equilibrium ratio
Nc: number of components
*x*_*i*_: composition of component *i* in phase *j*
*ω*_*i*_: acentric factor
*P*^sat^: saturated pressure, bar
*P*_c_: critical pressure, bar
*T*_c_: critical temperature, K
*T*: temperature, K
*z*_*i*_: mole fraction of component *i* in the overall mixture
